# Deep Brain Stimulation in Parkinson's Disease

**DOI:** 10.1155/2018/9625291

**Published:** 2018-02-07

**Authors:** Raja Mehanna, Hubert H. Fernandez, Aparna Wagle Shukla, Jawad A. Bajwa

**Affiliations:** ^1^University of Texas Health Science Center, Houston, TX, USA; ^2^Center for Neurological Restoration, Cleveland Clinic, Cleveland, OH, USA; ^3^University of Florida, Gainesville, FL, USA; ^4^Parkinson's, Movement Disorders and Neurorestoration Program, National Neuroscience Institute, King Fahad Medical City, Riyadh, Saudi Arabia

Deep brain stimulation (DBS) of the subthalamic nucleus (STN) and globus pallidus interna (GPi) is considered an essential therapy in the management paradigm of Parkinson's disease (PD). Its success stems essentially from its remarkable efficacy and, compared to the lesions created with thalamotomy and pallidotomy, its flexibility through programming that allows modification of the stimulation delivered to the precise brain targets, thereby obtaining maximal benefit with minimal side effects. In light of the impressive pace of advances in DBS technology, relentless exploration of new targets, development of programming paradigms, and continued controversy on patient selection and timing of DBS surgery, a special issue on this fascinating topic is pertinent and timely.

The choice of the DBS target between STN and GPi is driven by the constellation of motor and nonmotor symptoms which are key determinants to quality of life. U. Yazdani et al. discuss the pros and cons of each target and emphasize the need for these considerations while determining the final choice in a given individual. A widely accepted notion is that GPi DBS causes less cognitive decline than STN DBS and can be considered as the preferred target in PD patients with preoperative cognitive impairment. R. Mehanna et al. reevaluate this issue in a review of 72 studies totaling 2,410 STN DBS patients and 702 GPi DBS patients and draw a set of recommendations regarding the cognitive impact of DBS in PD patients.

While STN and GPi are the main targets of DBS in PD patients as they alleviate a broader spectrum of motor symptoms, in contrast to Vim which helps primarily with tremors [1], the benefit of their stimulation on axial and nonmotor symptoms is limited [2]. D. Anderson et al. describe the role of emerging alternative DBS targets such as the pedunculopontine nucleus, caudal zona incerta, substantia nigra pars reticulata, and the motor cortex for control of axial symptoms such as freezing, postural instability, gait, speech, and swallowing and nonmotor symptoms such as memory impairment, attention decline, and sleep disturbances in PD patients. Although initial reports are promising, carefully designed and larger controlled studies are required to verify the efficacy of these alternative DBS targets.

In addition to appropriate patient screening and target selection, careful programming is critical for a positive clinical outcome. Although general guidelines for DBS programming are available, a systematic protocol is lacking. Programming can thus be challenging, time-consuming, and labor-intensive. Nevertheless, with the advent of technological improvements, programming algorithms are expected to become more effective and less frustrating. A. Wagle Shukla et al. review the current approaches to DBS programming and summarize the most recent advances in the DBS field, including interleaving of DBS pulses, fractionated current, directional steering of current, use of biphasic DBS pulses, and closed-loop stimulation. The authors also discuss the role of computer-guided programming and the possibility of remote Internet-based programming which are promising approaches to impact access to DBS care in the near future.

Although the clinical efficacy of DBS in PD is well established, its mechanism of action is still partially understood but is being actively explored, especially in animal studies [3]. There are limited data on the impact of DBS on cognitive and emotional traits, the efficacy of unipolar versus bipolar stimulation, and the long-term sustainability of symptom alleviation after the cessation of DBS [4]. These are all addressed in an original article from K. Badstuebner et al.

In 2013, the EARLY-STIM trial provided Class I evidence for the use of DBS earlier in PD [5]. This finding led to the 2016 FDA approval of DBS in patients with at least 4 years of disease duration and 4 months of motor complications as an adjunct therapy for patients not adequately controlled with medications. A review from G. Suarez-Cedeno et al. highlights the changes overtime in DBS implantation, its current application, and the challenges that come with earlier intervention such as selecting appropriate candidates, predicting outcome, and managing side effects. The authors also discuss the current knowledge of the impact of DBS on mortality and its possible neuroprotective effects.

However, in a survey of 23 Swedish patients interviewed at a mean of 8 years after diagnosis, M. Sperens et al. report that patients in moderate stages of PD seem to be resistant to an earlier intervention. Despite the EARLY-STIM trial, a significant subset of patients still consider DBS as a last resort procedure. According to the authors, patients in their cohort had a reasonable perception of DBS, expressing caution “and well considered attitudes towards its outcome.” Their resistance to early intervention is thus not stemming from inappropriate concern or relative ignorance.

On the other hand, in a retrospective study of 29 patients, K. LaFaver et al. report that while all patients are at least partially content to have undergone DBS and will recommend it, only one-third feel that their preoperative education was *very adequate*, an additional 46% rate it as *adequate*, while 3.6% find it to be *completely inadequate*. This study underscores the need for a better preoperative education in order to insure realistic expectations and successful DBS outcomes [6].

Finally, determining predictors of functional and quality of life outcomes after DBS in PD would be useful to better tailor treatment to the individual patient's needs. H. Abboud et al. report on a retrospective review of pre- and postoperative data in 130 patients, suggesting that postural instability and worse pre-DBS motor score are the strongest predictors of poorer functional and QOL outcomes, while age at surgery and duration of the disease did not seem to influence the outcome. On the other hand, the presence of tremors and the absence of dyskinesia and of freezing of gait are reported as the greatest predictors of global improvement, confirming prior reports [7, 8]. This study is also the first to report preoperative high BMI as a potential predictor of poorer functional outcome. Furthermore, many reports suggested the possibility of significant weight increase after DBS in advanced PD, creating some concern among patients, especially in cases of obesity, diabetes, and other metabolic disorders [9]. It is thought to be secondary to reduction in the metabolic rate after resolution of tremor and/or dyskinesia and/or a direct stimulation effect on appetite centers [10–12]. In an original article, S. H. Millan et al. report different findings in early PD and offer insightful explanation to the difference. A phase III randomized controlled trial is underway and will look further into this question.

In summary, this special issue provides an updated overview of the ever-expanding field of DBS for PD, while probing into emerging and controversial themes. It compares and reevaluates effectiveness of STN versus GPi DBS targets on motor and nonmotor symptoms, objectively analyzes the available data on the impact of other potential targets, and challenges the conventional wisdom on factors that predict better outcome, optimal timing of surgery, and the appropriateness of patients, including their perceptions of the procedure prior to DBS surgery.

*Raja Mehanna* *Hubert H. Fernandez* *Aparna Wagle Shukla* *Jawad A. Bajwa*
